# Place of Death of People With Chronic Conditions in Latin America: A Systematic Review

**DOI:** 10.3389/phrs.2026.1609006

**Published:** 2026-03-05

**Authors:** María Adelaida Cordoba-Nuñez, Alejandro Unda-López, Paula Hidalgo-Andrade, Luis Fernando Rodrigues, Fernando Cesar Iwamoto Marcucci, Tania Pastrana

**Affiliations:** 1 Hospital Universitario de la Fundacion Santa Fe de Bogota, Bogotá, Colombia; 2 Department of Pediatrics, School of Medicine, Universidad de Los Andes, Bogota, Colombia; 3 Grupo de Investigación Bienestar, Salud y Sociedad, Escuela de Psicología y Educación, Universidad de Las Américas (UDLA), Quito, Ecuador; 4 Palliative Care Unit, Barreto’s Cancer Hospital, Barretos, Brazil; 5 Paraná State Health Secretariat, Hospital Dr. Anísio Figueiredo – Zona Norte de Londrina, Londrina, Brazil; 6 Department of Palliative Medicine, Uniklinik RWTH Aachen, Aachen, Germany; 7 International Association Hospice and Palliative Care, Houston, TX, United States

**Keywords:** end-of-life, healthcare system, Latin America, mortality, place of death, chronic condition

## Abstract

**Objective:**

To identify the place of death and associated factors among individuals in Latin American countries, with a particular focus on chronic conditions and serious health-related suffering.

**Methods:**

A systematic review was conducted across five databases in May 2024 following PRISMA guidelines. Eligible studies included data on the place of death of at least one Latin American country.

**Results:**

Twenty-one studies with data from 12 Latin American countries were included. Hospital was the most frequent place of death in Argentina, Brazil, Colombia, Costa Rica, Paraguay, and Uruguay, while home deaths predominated in Ecuador, El Salvador, and Guatemala. In Chile, Peru, and Mexico, the distribution was mixed, varying by population and clinical condition. Findings showed that older individuals and lower education were associated with an increased likelihood of home death. Marital status and urban residence showed mixed associations. A meta-analysis was not feasible due to high heterogeneity among the studies.

**Conclusion:**

The place of death of people with chronic and serious health-related conditions in Latin America varies considerably, reflecting disparities in healthcare access, sociocultural values, and health system infrastructure. Findings highlight the need for country-specific, equity-oriented end-of-life care policies.

**Systematic Review Registration:**

https://www.crd.york.ac.uk/prospero/display_record.php?ID=CRD42024553349.

## Introduction

The place of death (PoD) is considered an indicator of the quality of end-of-life care, particularly among individuals with a chronic condition who require palliative care [1]. Given this relevance, the World Health Organization (WHO) has identified the PoD as a public health priority since the possibility of choosing the PoD reflects health system responsiveness [2]. In addition to the ethical responsibility of health systems to alleviate pain and suffering at the end of life [3], PoD also carries significant economic implications [4], particularly given the high costs of end-of-life care in hospitals [5–7].

While PoD has been more extensively studied in high-income countries [1, 2], its determinants remain poorly understood in Latin America. Accounting for 8.4% of the global population, Latin America is undergoing demographic and epidemiological transitions that are expected to increase the burden of Serious Health-related Suffering (SHS) in the coming decades [8]. These transitions are likely to amplify the economic and social impact, which might vary depending on the location where care is provided [9]. Although all countries in the region offer some form of palliative care, access remains heterogeneous, reflecting inequities within national healthcare systems [10]. These disparities, along with the need for culturally sensitive services, complicate the provision of end-of-life care in the region [11].

Previous research has identified associations between PoD and several sociodemographic, clinical, and environmental factors, including sex, age, marital status, socioeconomic status, and underlying disease. Understanding how these factors affect PoD is essential for identifying inequity and optimizing end-of-life care planning to ensure that services align with patients’ needs and pRefs. [1, 12].

PoD data is typically derived from national civil registration and vital statistics (CRVS) systems, which include information from death certificates and other official records. However, the quality, completeness, and standardization of CRVS systems vary widely across Latin American countries [13].

This systematic review aims to consolidate existing evidence on the PoD of people with chronic conditions in Latin America, shedding light on cultural, socioeconomic, and healthcare system factors that influence the PoD in the region. Specifically, we address the following research questions: 1) Where do people with chronic conditions experiencing serious health-related suffering die in Latin America? and 2) What are the factors associated with the PoD among individuals with chronic conditions in Latin America? In this review, we use the term *chronic conditions* to refer to long-term, progressive, and life-limiting diseases commonly associated with serious health-related suffering, including HIV/AIDS, cancer, advanced organ failure, neurodegenerative disorders, and other non-communicable conditions [14].

## Methods

This systematic review explored the PoD in Latin America, following the Joanna Briggs Institute (JBI) methodological recommendations for systematic reviews [15]. The review adhered to the PRISMA 2020 reporting guidelines, and the protocol was registered in PROSPERO (ID: CRD42024553349) [16].

### Search Strategy and Selection Criteria

The literature search was performed in May 2024 across five databases: EMBASE, MEDLINE, PsycINFO, LILACS, and SciELO. The search strategy included terms related to ‘place of death’ and ‘Latin America’ and was designed without restrictions on population, study type, publication date, or language. It was adapted for each database as appropriate. For LILACS and SciELO, the strategy included translations of key terms into Spanish and Portuguese to maximize sensitivity. Furthermore, a manual search of the reference lists of relevant studies was conducted to identify additional sources.

The final query string used in the selected databases was: (Latin America OR “South America” OR “Central America” OR Argentina OR Bolivia OR Brazil OR Chile OR Colombia OR “Costa Rica” OR Cuba OR Ecuador OR “Dominican Republic” OR “El Salvador” OR Guatemala OR Honduras OR Nicaragua OR Mexico OR Panama OR Paraguay OR Peru OR Uruguay OR Venezuela) AND ((place OR location OR site) AND death) OR ((place OR location OR site) AND dying). For the full query strings used in each database, please refer to Supplementary Material 1. Eligible studies included primary quantitative studies reporting PoD in at least one Latin American country. Studies explicitly focused on deaths from chronic or life-limiting conditions—such as cancer, dementia and other neurodegenerative diseases, chronic respiratory or cardiovascular disease, and HIV/AIDS—were prioritized. Studies focusing solely on preferred PoD, deaths occurring outside Latin America, or lacking sufficient detail on PoD were also excluded. Grey literature was not included.

Population-based studies using all-cause mortality data were included when they reported PoD at the national or regional level and when cause-of-death information or stratified analyses allowed interpretation relevant to chronic conditions or end-of-life care. Studies exclusively focused on acute causes of death (e.g., trauma, accidents, violence, obstetric complications, or sudden external causes) were excluded.

### Selection Process

**FIGURE 1:**
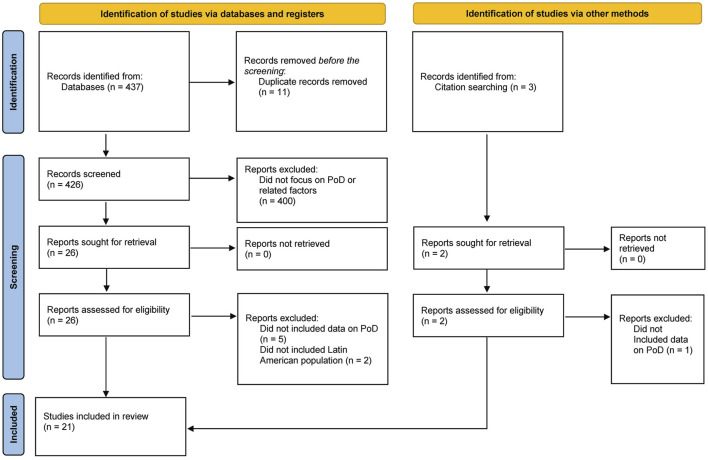
Flow diagram of included studies according to PRISMA guidelines [18], (Latin America, 2024).

All the references were imported and managed using Covidence software [17]. Two reviewers independently screened all titles and abstracts to establish eligibility for full-text review and remove irrelevant studies. Discrepancies at any stage were discussed in research sessions until a consensus was reached. Full-text copies of all relevant articles were then retrieved and independently assessed by two reviewers against the inclusion/exclusion criteria. Disagreements during full-text screening were resolved similarly through consensus. All exclusion reasons were noted and provided in the PRISMA diagram (Figure 1).

### Data Extraction

Two researchers processed data independently using a pretested data extraction form. Extracted information included author, year of publication, study aims, design, country of study, data source, population, missing values, eligibility criteria, exclusion criteria, and information sources.

For primary outcomes, data were extracted on sociodemographic variables (e.g., age, sex, marital status), socioeconomic factors (e.g., education, urban/rural residence), healthcare system-related factors (e.g., hospital bed density), cause of death, and proportion/frecuency of deaths by PoD. Places of death included home, hospital, and other facilities (the term “other facilities” was used to account for variability in the operational definitions of non-home and non-hospital PoD across the included studies). Healthcare system-related factors were classified as variables related to health service delivery, such as availability of hospital beds, distinct from individual-level sociodemographic or socioeconomic factors.

### Quality Assessment and Grading of Evidence

We conducted a quality assessment of the included studies using the approach from previous reviews of PoD and associated factors [19]. This approach is based on the Strength of Recommendation Taxonomy (SORT) [20] and applied to PoD by Gomes et al. [19]. This tool evaluates methodological aspects of observational studies, including study design, data source reliability, sample size, and risk factor analysis, classifying evidence as low, medium, or high quality [19–21]. Quality appraisal score was attained by calculating the percentage of the sum of the scores for each item in relation to the maximum possible score [20]. The interpretation was based on SORT tool [19] method of grading (High quality ≥70%; medium quality ≥60%; low quality <60%). Two researchers independently applied the scale in Covidence, scoring each study on criteria including representativeness, data completeness, and adjustment for confounders. Discrepancies were resolved through team discussion.

### Data Analysis

We conducted a descriptive synthesis of the included studies, focusing on patterns related to PoD and associated factors. The primary outcome was frequency of deaths occurring at home, in hospitals, or in other settings, reported as both absolute numbers and percentages across countries and populations.

We also identified and categorized factors influencing PoD, including sociodemographic, clinical, and environmental factors. For each factor, we recorded the direction of its association (i.e., whether it increased the likelihood of dying at home or in a hospital) and graded the strength and quality of supporting evidence. Descriptive statistics were used to identify general patterns and trends. We reported the odds ratios (OR) with 95% Confidence Interval (CI) provided by the studies.

A meta-analysis was planned in accordance with Cochrane guidelines to individually explore the OR of sex, age, marital status, education, place of residence, and cause of death on PoD, with only studies of high methodological quality being considered for inclusion. An exploratory analysis using a random-effects model was conducted to address expected heterogeneity. Studied factors were eligible if examined in at least five studies; however, an initial assessment revealed substantial heterogeneity across the included studies (I^2^ > 90%). The presented heterogeneity may be attributed to a considerable diversity between sample characteristics, sample sizes, data collection moments, the extended period for data collection between included studies (1990–2021), and the operational definitions used for each PoD and associated factors (e.g., “urban” and “rural” as categories for the classification of place of residence). Therefore, following methodological guidelines [22], further analysis was not conducted to avoid introducing bias and compromising the validity of the findings.

## Results

After removing duplicates, a total of 440 studies were identified for initial screening through database and citation searching. Of these, 21 met the inclusion criteria and were included in the final data charting, encompassing patient data from 12 Latin American countries. However, some studies drew from the same or overlapping databases. For instance, Cárdenas-Turanzas et al. used 2003 data from the Mexican Ministry of Health (‘Secretaría de Salud’), while Cárdenas-Turanzas et al. used data from 2002 to 2004 from the same source. For specific information on screening, refer to the PRISMA flow diagram [23] in Figure 1.

### Overview of the Studies

Most studies (n = 12) focused on populations from Mexico [12, 24–34], followed by Brazil (n = 7) [24, 35–40], Chile (n = 5) [24, 40–43], and Peru (n = 2) [24, 40]. Additionally, data from Argentina, Colombia, Costa Rica, Ecuador, El Salvador, Guatemala, Paraguay, and Uruguay were reported in a single country study that included 12 Latin American countries [24].

The earliest dataset was collected in 1990 in Chile [41, 42], while the most recent data was from 2021 [37, 40]. The included studies reported diverse designs: Four studies reported a retrospective approach [12, 25, 32], three a cross/sectional design [28, 37, 42], two a time series analysis [40, 43], two a population level design [29, 31], one an observational design [24], and one an ecologic descriptive exploratory design [38]. Six studies did not report explicitly the study design implemented [26, 27, 30, 34, 36, 41]. In practice, all studies used data collected in a single year, except for two that used data over several years for time-series analysis [40, 43].

Regarding data sources, 11 studies relied on death certificates [12, 26–33, 37, 41], eight studies used national mortality data [34–36, 38–40, 42, 43], which typically consist of information gathered by government or international agencies in each country, differing in the data collection, refinement, and reporting processes from death certificates alone. One study used both death certificates and national mortality data [24], while another used information from a health and aging survey [25].

The quality assessment showed that most included articles had high (12 out of 21 studies) or medium (5 out of 21 studies) quality, except for four articles that received low scores [35, 39–41]. The complete charting of study characteristics is presented in Table 1.

**TABLE 1:** Overview of study characteristics (Latin America, 2007–2024).

First author and year of publication	Countries where data was collected	Setting	Study design (as stated by the authors)	Data source	Study period (start - end)	Number of participants in the study	Quality appraisal score (%)
Seitz [24]	Argentina; Brazil; Chile; Colombia; Costa Rica; Ecuador; El Salvador, Guatemala; Mexico; Peru; Paraguay; Uruguay	Country	Observational	Death certificates; national mortality data	2016–2018	2994685	83.3
Furukawa [38]	Brazil	Region	Ecological descriptive exploratory	National mortality data	2007–2007	5834	70.8
Leite [39]	Brazil	City	Descriptive	National mortality data	2006–2012	63343	58.3
Lopes da Silva [37]	Brazil	Country	Cross-sectional	Death certificates	2002–2021	3677415	81.3
Marcucci [35]	Brazil	City	Descriptive	National mortality data	1996–2010	57768	35.4
Marcucci [36]	Brazil	Country	Not reported	National mortality data	2002–2013	12974742	68.8
Durán [40]	Brazil; Chile; Peru	Country	Interrupted time series analysis	National mortality data	2019–2021	3753804	58.3
Browne [42]	Chile	Country	Cross-sectional	National mortality data	1990–2014	2063615	81.3
Leiva [41]	Chile	Region	Not reported	Death certificates	1990–2003	1102896	58.3
Paredes [43]	Chile	Country	Time series study	National mortality data	1997–2014	1576306	77.1
Cárdenas-Turanzas [12]	Mexico	Region	Retrospective	Death certificates	2003–2003	10561	75.0
Cárdenas-Turanzas [33]	Mexico	Region	Retrospective	Death certificates	2002–2004	1948	77.1
Cárdenas-Turanzas [25]	Mexico	Country	Retrospective	Mexican health and aging study	2001–2003	473	72.9
Castillo-Guzmán [34]	Mexico	Country	Not reported	National mortality data	1999–2009	5338322	68.8
Cohen [28]	Mexico	Country	Cross-sectional	Death certificates	2008	65812	77.1
Cohen [29]	Mexico	Country	Population-level	Death certificates	2005–2006	27647	72.9
Håkanson [31]	Mexico	Country	Population-level	Death certificates	2008	4653	81.3
Harding [32]	Mexico	Country	Retrospective	Death certificates	2008	5149	79.2
Moens [27]	Mexico	Country	Not reported	Death certificates	2008	1062	81.3
Pivodic [30]	Mexico	Country	Not reported	Death certificates	2008	131013	81.3
Reyniers [26]	Mexico	Country	Not reported	Death certificates	2008	2060	81.3

### Place of Death

At the country level, hospitals were the most frequent PoD in Argentina (69.5%), Brazil (65.9%–88.2%), Colombia (64.7%), Costa Rica (53.7%), Paraguay (58.6%), and Uruguay (53.6%). Regional analysis within Brazil further confirmed this trend [35, 38, 39]. In contrast, in Ecuador (48.9%), El Salvador (50.7%), and Guatemala (67.9%), home death was more common.

Two studies reported minimal differences (less than 5%) between hospital and home deaths in the general population in Mexico [24, 25]. However, six national studies found that home deaths were more common among adults with cancer and chronic conditions [26–30, 34]. This trend was further supported by a study in Mexico City showing a higher prevalence of home deaths among adults aged 18 and older with cancer [12]. In contrast, two studies reported a higher prevalence of hospital deaths among children [31, 34], a finding reinforced by a focused analysis conducted in Mexico City [33]. Additionally, one national study found that hospital deaths were more frequent among people living with HIV [32].

In Chile, two studies identified home as the most frequent PoD [24, 40]. However, another study found a higher prevalence of in-hospital mortality among adults aged 85 and older. PoD for cancer patients was predominantly outside the hospital, with no substantial change in trend over the analyzed period [42]. Another study reported similar frequencies of hospital and home deaths, aligning with the national trend [43]. Data from a regional study in Chile observed a slight increase in hospital deaths, particularly among children and adults under 60 years old in this region. The authors of that study attributed this pattern to the limited availability of home-based care services in that area during the study period [41]. The results are shown in Table 2.

**TABLE 2:** Distribution of place of death by country, context, and population characteristics in Latin America (Latin America, 1990–2021).

First author and year of publication	Country and context of data collection	Sample characteristics	Additional study characteristics	Home death (%)	Hospital death (%)	Other (%)
Seitz [24]	Argentina Country	>1 year	N/A	22.9	69.5	7.6
Marcucci [35]	Brazil City	N/A	1996	16.1	76.1	7.9
Marcucci [35]	Brazil City	N/A	2010	17.4	74.1	8.6
Leite [39]	Brazil City	Cancer; >60 years	N/A	9.0	88.2	N/A
Furukawa [38]	Brazil Region	>45 years	N/A	27.0	73.0	0.0
Lopes da Silva [37]	Brazil Country	>20 years	N/A	17.7	82.3	N/A
Marcucci [36]	Brazil Country	>60 years	2002	23.5	66.7	1.5
Marcucci [36]	Brazil Country	>60 years	2013	19.8	66.7	4.2
Durán [40]	Brazil Country	Malignant neoplasms	N/A	19.1	75.4	5.6
Seitz [24]	Brazil Country	>1 year	N/A	20.0	65.9	14.1
Leiva [41]	Chile Region	Children	Rest of the country	46.6	44.8	8.6
Leiva [41]	Chile Region	Children	Sixth Region	44.3	46.0	9.7
Browne [42]	Chile Country	>1 year	N/A	N/A	43.6	56.4
Paredes [43]	Country	>1 year	N/A	46.2	44.2	9.6
Durán [40]	Chile Country	Malignant neoplasms	N/A	47.4	45.6	7.0
Seitz [24]	Chile Country	>1 year	N/A	49.7	40.0	10.4
Seitz [24]	Colombia Country	>1 year	N/A	26.5	64.7	8.8
Seitz [24]	Costa Rica Country	>1 year	N/A	37.1	53.7	9.2
Seitz [24]	Ecuador Country	>1 year	N/A	48.9	42.9	8.2
Seitz [24]	El Salvador Country	>1 year	N/A	50.7	35.4	13.9
Seitz [24]	Guatemala Country	>1 year	N/A	67.9	22.3	9.9
Cárdenas-Turanzas [12]	Mexico Region	>18 years	N/A	54.0	46.0	N/A
Cárdenas-Turanzas [33]	Mexico Region	Children with cancer	N/A	15.0	85.0	N/A
Cárdenas-Turanzas [25]	Mexico Country	Adults born before 1951	N/A	52.9	47.1	N/A
Reyniers [26]	Mexico Country	Dementia-related deaths	N/A	69.3	26.2	4.5
Moens [27]	Mexico Country	Parkinson’s	N/A	73.0	24.1	2.9
Cohen [28]	Mexico Country	Cancer	N/A	57.3	39.9	2.7
Pivodic [30]	Mexico Country	>1 year	N/A	53.0	44.4	2.7
Håkanson [31]	Mexico Country	>1 year	N/A	31.7	65.3	3.0
Cohen [29]	Mexico Country	Lung cancer and COPD	COPD	55.4	41.8	2.8
Cohen [29]	Mexico Country	Lung cancer and COPD	Lung cancer	57.1	40.1	2.8
Harding [32]	Mexico Country	HIV/AIDS	N/A	26.3	71.0	2.8
Castillo-Guzmán [34]	Mexico Country	Cancer; >85 years	N/A	55.7	39.1	5.3
Seitz [24]	Mexico Country	>1 year	N/A	47.0	44.0	9.1
Seitz [24]	Paraguay Country	>1 year	N/A	30.7	58.6	10.8
Durán [40]	Peru Country	Malignant neoplasms	N/A	40.2	55.4	4.5
Seitz [24]	Peru Country	>1 year	N/A	43.0	48.9	8.1
Seitz [24]	Uruguay Country	>1 year	N/A	39.1	53.6	7.2

This table uses a heat map to highlight the distribution of PoD percentages across studies. Higher values appear in red to indicate more frequent PoD in that setting, while lower values appear in green for easy comparison between studies.

### Associated Factors

Several individual-level and contextual factors were associated with PoD. Individual-level determinants included age, marital status, educational attainment, and cause of death, while contextual and health-system-related determinants included place of residence and access to services. Table 3 summarizes these associations, their direction, and the quality of supporting evidence. A comprehensive synthesis of these factors, including direction and strength of association, statistical significance, number of supporting studies, and quality of evidence, is presented in Table 4.

**TABLE 3:** Factors associated with the place of death, according to the quality of evidence (Latin America, 1990–2021).

Factor	High quality of evidence		Moderate quality of evidence	
	Death more likely at	Sources of evidence	Death more likely	Sources of evidence
Older age^a^	Home	[25, 37, 38, 43]	Home	[34, 42]
Marital status	Contradictory findings	[24, 37, 43]	Contradictory findings	[36]
Residence urban	Contradictory findings	[24]	Contradictory findings	[34]
Non-cancer	Home	[24, 29, 31]	​	​
Hematological malignancies^b^	Hospital	[12, 33, 34]	​	​

^a^ Overall, a higher age was associated to home death, although age cut points vary across studies.

^b^ Primary Leukemia.

**TABLE 4:** Individual-level and contextual determinants associated with place of death in Latin America (Latin America, 1990–2021).

Determinant	Level	Direction of association (home vs. hospital death)	Strength of association (OR, 95% CI)	Statistically significant	Supporting studies (n)	Quality of evidence	References
Age (older age)	Individual	Home death more likely	OR range: 1.02–7.62	Yes	6	High	[33, 34, 37–39, 41, 42]
Marital status – Married vs. single	Individual	Contradictory	OR range: 0.84–1.15	Yes	3	Moderate–High	[24, 39, 43]
Marital status – Widowed vs. married	Individual	Contradictory	OR range: 0.93–1.38	Yes	2	Moderate	[24, 43]
Educational level – Low education	Individual	Home death more likely	OR range: 1.07–3.38	Yes	3	High	[37, 38, 43]
Educational level – Secondary or university	Individual	Hospital death more likely	OR: 0.36	Yes	1	Moderate	[34]
Cause of death – Non-cancer conditions	Individual	Home death more likely	Narrative	Yes	3	High	[24, 29, 31]
Cause of death – Solid tumors	Individual	Home death more likely	Narrative	Yes	3	High	[24, 29, 31]
Cause of death – Hematological malignancies	Individual	Hospital death more likely	Narrative	Yes	4	High	[12, 33, 34, 39]
Children (pediatric deaths)	Individual	Hospital death more likely	Narrative	Yes	3	High	[31, 33, 34]
Rural residence	Contextual/Health-system	Home death more likely	OR range: 1.39–2.53	Yes	3	High	[24, 34, 43]
Distance from major urban centers	Contextual/Health-system	Home death more likely	OR = 1.33	Yes	1	Moderate	[38]

Direction of association indicates whether the determinant was associated with a higher likelihood of home or hospital death. Strength of association is reported as odds ratios (OR) using the range reported in the studies; when quantitative estimates were not provided, associations are reported narratively. Quality of evidence was assessed using a Strength of Recommendation Taxonomy (SORT)-based approach adapted for place-of-death studies. Associations are derived from observational population-level studies and should not be interpreted as causal.

Direction of association indicates whether the determinant was associated with a higher likelihood of home or hospital death. Quality of evidence was assessed using a Strength of Recommendation Taxonomy (SORT)-based approach adapted for place-of-death studies. Associations are derived from observational population-level studies and should not be interpreted as causal.

### Age

Six studies found a positive association between advanced age and increased likelihood of home death. Specifically, studies found that being over 60 (OR = 1.66; 95% CI = 1.65–1.67) [37], over 65 (OR = 1.99; 95% CI = 1.7–2.33) [38], over 70 (OR = 1.02; 95% CI = 1.02–1.02) [43], over 75 (OR = 1.03; CI = 95% = 1.01–1.05) [33], over 85 (OR = 2.52; 95% CI = 2.49–2.55) [34, 42] and over 90 (OR = 7.62; 95% CI = 7.30–7.95) [39] were more likely to die at home. It is worth noting that most studies presented different reference points for age. Specific measures of association are reported in Table 5.

**TABLE 5:** Age associated with the place of death (Latin America, 1990–2021).

First author and year of publication	Age threshold	Odds ratio (OR)	95% CI
Lopes da Silva [37]	>60 years	1.66	1.65–1.67
Furukawa [38]	>65 years	1.99	1.70–2.33
Paredes [43]	>70 years	1.02	1.02–1.02
Cárdenas-Turanzas [33]	>75 years	1.03	1.01–1.05
Castillo-Guzmán [34]	>85 years	2.52	2.49–2.55
Leite [39]	>90 years	7.62	7.30–7.95

### Marital Status

The findings regarding marital status vary across the region. One study in Chile indicated that being married (OR = 1.15; 95% CI = 1.14–1.16) and being widowed (OR = 1.38; 95% CI = 1.37–1.40) had a higher likelihood of dying at home in comparison to single people [43]. However, when comparing being widowed to being married, being a widow was associated with a lower likelihood of home death (OR = 0.93; 95% CI = 0.89–0.97). In contrast, another study in Brazil found that married people had a lower likelihood of dying at home (OR = 0.84; 95% CI = 0.77–0.92) compared to those who were single [39]. A multicentric study compared eight Latin American countries, examining these associations using married individuals as the reference group, showed that being single, widowed, or divorced was consistently associated with a higher likelihood of home death in most countries studied [24]. The results of this multicenter study [24] are presented in Table 6.

**TABLE 6:** Marital status as a factor for increased likelihood of home death [24], (Latin America, 2016–2018).

Country	Divorced vs. Married Odds ratio (95% CI)	Married vs. Single Odds ratio (95% CI)	Single vs. Married Odds ratio (95% CI)	Widowed vs. Married Odds ratio (95% CI)	Widowed vs. Single Odds ratio (95% CI)
Brazil (2017)	1.24 (1.21–1.26)	0.84 (0.77–0.92)	1.37 (1.35–1.38)	1.13 (1.12–1.14)	​
Chile (2016)	​	1.15 (1.14–1.16)	​	0.93 (0.89–0.97)	1.38 (1.37–1.40)
Colombia (2017)	1.22 (1.16–1.28)	​	1.29 (1.26–1.33)	1.18 (1.14–1.21)	​
El Salvador (2017)	​	​	1.18 (1.12–1.24)	​	​
Mexico (2017)	​	​	1.48 (1.46–1.50)	1.17 (1.16–1.19)	​
Paraguay (2017)	​	​	1.24 (1.17–1.24)	1.22 (1.12–1.32)	​
Peru (2017)	1.39 (1.25–1.56)	​	​	1.52 (1.45–1.60)	​
Uruguay (2018)	​	​	​	1.2 (1.12–1.28)	​

### Education

Lower educational level was consistently associated with a higher likelihood of home death. Specifically, no formal education (OR = 3.38; 95% CI = 3.35–3.41) [37], less than 3 years of schooling [38] (OR = 1.7; 95%, CI = 1.33–2.18), or achieving only basic education (OR = 1.07; 95% CI = 1.06–1.08) [43] were more likely to die at home. In line with these findings, individuals with either secondary (OR = 0.36; 95% CI = 0.35–0.37) or university-level education (OR = 0.38; 95% CI = 0.37–0.39) were less likely to die at home [34].

### Place of Residence

Death in rural areas was generally more likely to occur at home, as reported in Colombia (OR = 1.39; 95% CI = 1.35–1.43), Ecuador (OR = 1.52; 95% CI = 1.46–1.58), and El Salvador (OR = 2.53; 95% CI = 2.42–2.65) [24]. The same trend was confirmed in Mexico by three separate studies [24, 34, 43].

While not a direct indicator of urbanization, one study in Brazil found that residing in municipalities far from the main city in the region increases the likelihood of dying at home (OR = 1.33; 95% CI = 1.18–1.50) compared to cities with a high rate of urbanization [38].

### Cause of Death

The underlying cause of death was also associated with the PoD. Individuals with non-cancer conditions or with solid tumors were more likely to die at home. Three high-quality studies found it more likely to die at home if the cause of death was non-cancer; this was also observed in children [24, 29, 31]. In contrast, death due to hematological malignancies was consistently associated with a higher likelihood of hospital death, compared to those due to solid tumors [12, 33, 34, 39].

## Discussion

### Summary of Main Findings

This systematic review aimed to consolidate existing evidence on the PoD of people with chronic conditions in Latin America, shedding light on cultural, socioeconomic, and healthcare system factors that influence the PoD in the region. The PoD is a public health priority because it influences the quality of death and the costs to the healthcare system [2]. It also indirectly reflects how well a health system supports people’s values and needs at the end of life. This systematic review included 21 peer-reviewed manuscripts on PoD in Latin America, of which 57% were deemed high-quality studies.

The evidence only includes 12 countries in the region, and the findings reveal that PoD trends vary significantly. For instance, hospital deaths are more frequent in Argentina, Colombia, Brazil, Costa Rica, and Uruguay. These countries often have more developed healthcare systems and more accessible hospital facilities in urban areas [44, 45]. Conversely, for Ecuador, Mexico, El Salvador, and Guatemala, the most common site of death was at home. Cultural attitudes toward dying and death may significantly influence PoD; in some Latin American countries, there may be a strong cultural preference for dying at home surrounded by family, aligning with traditional values that emphasize familial support and care [46, 47].

### Factors Influencing Place of Death

This section delves on the sociodemographic factors that may be linked to PoD in this region. Given the socioeconomic context in many countries, these associations should be explored with an equity lens. Age was a consistent factor, with older individuals showing a higher chance of dying at home. These results align with existing literature [48], which suggests that older adults may be more likely to accept death at home and are often perceived as appropriate candidates for non-institutional end of life. Conversely, younger individuals, particularly children, often die in hospital settings [49]. This trend may reflect a tendency towards more aggressive life-extending treatments, frequently requiring hospitalization in the pediatric population [50]. A clinical consideration is the patient’s performance status; children with advanced illnesses often require intensive medical support that may not be feasible at home due to limited resources. In such cases, hospitalization is seen as the only option for palliative care, especially in rural areas where geographic gaps in service coverage restrict access to home-based care, leading to urban migration in search of care [51]. As a result, despite families’ preferences for home death, hospital-based care becomes the only choice [52]. An additional contributing factor is the delayed integration of pediatric palliative care, resulting from systemic challenges such as limited availability of home-based services, a lack of trained personnel, and insufficient awareness among healthcare providers and families [53]. Trends in the PoD of children should be analyzed separately, because mortality is concentrated at the extremes of age. However, pediatric and adult deaths differ in underlying causes, care trajectories, and typical places of death.

Findings on marital status were heterogeneous across countries. For instance, in comparison with married people, being single or divorced is associated with increasing odds of home death [24]. Similarly, in Brazil, a study found that being married reduces the odds of dying at home in comparison with being single [39]. In Chile, being married or widowed increases the odds of dying at home, although widowed people had lower odds than those who were married [43]. This variability in the results may be attributed to other factors that interact with marital status, such as age, cultural background [54], and access to various resources, including informal care and community-based care [55]. This is especially relevant in Latin America, where multigenerational living arrangements and extended family support are common in many countries. Married individuals often reside in households that include spouses, children, and other relatives; this structure reinforces mutual care and family cohabitation [56]. Single individuals, especially those in rural areas or vulnerable situations, may live with relatives rather than alone due to economic and practical constraints [57]. Family networks often ensure that married older adults remain in their family homes or under the care of direct relatives rather than living alone or in institutions. In addition, widowed women in Latin America tend to reside with family members, such as adult children, due to cultural norms that prioritize family care [57].

A factor contributing to extended family involvement in end-of-life care is the sense of social obligation [47]. Family members, usually women, often assume caregiving roles, whether out of love for their relatives and a desire to give back for the care they once received, mainly when supporting aging parents. This sense of duty and emotional connection reinforces the tradition of family-centered care during the end of life [11]. Although extended families have historically been dominant in the region, social transitions such as urbanization, migration, and changes in cultural values are gradually increasing the number of people living alone, even among those previously married. We hypothesize that the civil status as recorded on the death certificate is an imperfect proxy for the deceased’s living arrangements and does not reliably indicate whether the deceased lived alone or had access to informal or familial support networks [58].

Other factors, such as educational attainment and place of residence, were less studied and showed differences between countries. The level of education is often considered a proxy for socioeconomic status in research and policy analysis. Studies evaluating the effect of the educational level on PoD show that achieving only basic education is related to an increased probability of home death [24]. Conversely, people who had achieved preparatory or university-level education showed a decreased likelihood of dying at home [19]. This highlights an inverted social gradient in PoD that operates in the opposite direction to that observed in Western Europe and North America, where resources such as wealth, knowledge, and social connections are often utilized to facilitate dying at home. In contrast, in many Latin American countries, these same resources are leveraged to secure access to hospital care at the end of life, and a better ability to navigate complex medical systems. Access to resources and healthcare may mediate the relationship between education and PoD, with higher education potentially correlating with better access to institutional care; and, in contrast, lower education levels may align with limited access to healthcare facilities, making home death more common not by preference, but by circumstance, likely in the absence of adequate care provision [59].

Residence area was also deemed an influential factor, as residing in less populated or rural areas was associated with an increased likelihood of dying at home [24, 25, 34, 38, 43]. This trend is likely driven by limited access to health-related resources in rural areas [46] and not specifically a decision for home as PoD. Furthermore, these trends may reinforce lower health literacy in rural areas, limiting the knowledge of available services, the ability to navigate the healthcare system, or the means to afford costs [60], further reinforcing non-institutional deaths by default rather than by choice.

Both factors, level of education and place of residence, appear to be connected, as they are associated with limited access to healthcare resources and with lower household income, particularly among economically disadvantaged populations. A factor often overlooked in studies is family income, which can significantly influence healthcare access and decision-making. Additionally, informal healthcare expenses, such as out-of-pocket costs for medications, transportation, and home care, exacerbate disparities, disproportionately affecting low-income families, and limiting their ability to seek adequate medical care [61]. Data on the causes of death were scarce in the reviewed studies; therefore, future studies could place greater emphasis on the inclusion of diagnostic and comorbidities to better understand the context around the death as a consequence of a specific condition. Additionally, this lack of diagnostic detail limits the ability to compare the influence of specific health conditions on PoD.

### Policy Implications and Future Research Priorities

Considering the influence that social factors have on end-of-life care, public policies should be strengthened to expand access to professional healthcare and proper death registration for the most socially vulnerable population. Underreporting and misclassification are more common in rural and underserved areas, particularly for deaths occurring outside of health facilities. The person responsible for certifying PoD varies depending on circumstances, ranging from hospital physicians to family members or civil authorities in the event of community deaths, which can affect data reliability [62]. These inconsistencies may introduce misclassification, selection bias, and urban–rural disparities. For instance, between 15% and 20% of rural deaths in Colombia go unregistered in areas with limited healthcare access, a trend also reported in Mexico [63, 64].

Additionally, the results regarding factors associated with PoD of people with chronic and HRS conditions suggest that PoD is a marker of unmet needs and social inequality. Thus, policy and investment should aim to transform the underlying factors that limit access to both institutional and home-based support, such as integrating palliative care into primary healthcare in rural and urban areas and creating caregiver support laws that address the burden of informal caregiving and promote trained and supported care.

Finally, there is a need to strengthen the evidence used for decision-making and research. Healthcare professionals should be properly trained to provide community-based end-of-life care, and research should opt to maintain standardized methodologies to minimize heterogeneity and facilitate meaningful cross-country comparisons. This includes developing or adapting indicators of quality of care and indicators to distinguish between planned home or institutional death, as well as possible consequences of a lack of access to resources or proper care. Additionally, it is essential to include intersectional data and underrepresented countries and populations to promote a more comprehensive understanding of PoD across Latin America’s diverse sociocultural and healthcare contexts. Moreover, existing literature would benefit from including information on the place of care and PoD preference. In addition, longitudinal and qualitative research are also needed to capture the cultural and contextual nuances that influence PoD. These efforts will provide more accurate, equible and regionally relevant insights to inform policy and practice.

### Limitations

This review has several limitations. The data reviewed and presented were not standardized or adjusted for inter-study comparisons, and several studies did not report having considered cofounding variables in their analyses. This may cause an overestimation of reported results. High heterogeneity between studies may diminish the ability to compare associations; therefore, accounting for generalization and causal inferences is not possible. Research in Latin America regarding end-of-life care could benefit from more replicable and transparent studies, as well as from a wider use of standardized and validated data collection tools.

On the other hand, the quality and completeness of CRVS systems across Latin America influence the accuracy and comparability of PoD data. Although death certificates served as the foundational source, national mortality datasets vary in how information is accessed, processed, and categorized. Variations in coding practices, inclusion criteria, and the classification of PoD may affect comparability across studies. While most countries require the inclusion of PoD in death certificates, the implementation and enforcement of these systems vary substantially. Countries such as Chile, Uruguay, and Costa Rica have relatively robust death registration systems, characterized by high data completeness. In contrast, other countries, including Honduras, Bolivia, and Guatemala, experience challenges related to underreporting [13]. Previous research has noted that the validity of PoD as an indicator of access to palliative care depends largely on the robustness of national CRVS systems [24].

The association between cause of death and PoD has been scarcely studied in the region. Most studies focused on a few countries, such as Brazil and Mexico, leaving many others underrepresented. As a result, the regional generalizability of the findings is limited. Nevertheless, these two countries account for more than half of Latin America’s population, which may partially explain their overrepresentation in PoD studies. Most studies provided diagnostic information only for cancer-related deaths, preventing comparisons across other conditions (e.g., dementia or heart failure). Additionally, the analysis of sociodemographic factors such as age, education, and marital status was often conducted without adjustment for covariates or (potential) interactions between variables, which could lead to oversimplified interpretations of complex relationships and to incorrectly estimating the effect of an isolated factor.

Furthermore, most studies lacked data on modifiable determinants of PoD, such as timely access to health services, palliative care coverage, or availability of essential medications. These structural factors are crucial to understanding disparities in end-of-life care, but they are rarely captured in administrative datasets. Finally, the methodological rigor and data quality varied across studies. As the specific sample size and characteristics used in each study are not reported, results from these manuscripts should be interpreted with caution, as the pooled sample may include duplicated data. In addition, it is worth noting that the included data were collected from 1990 to 2021, a period that encompasses the COVID-19 pandemic. As this review does not account for temporal variability, future studies should include a time-series analysis to detect time-related influences on factors associated with PoD.

## Conclusion

PoD in Latin America exhibits significant variability in both distribution and associated factors. Hospitals were the predominant PoD in most of the included Latin American countries (6/12), while some report a higher frequency of home deaths (3/12) and others (3/12) show mixed results. This may reflect disparities in health system accessibility, cultural preferences, and resource availability. Factors such as age, education level, marital status, and cause of death influence PoD, yet the direction and magnitude of these associations vary across contexts. Other key determinants, such as residential settings, household income, informal care costs, and comorbidities, remain understudied and yield contradictory findings, necessitating further investigation to clarify their role in PoD. Moreover, mortality patterns and PoD among children differ considerably from those of adults; therefore, disaggregated analyses are necessary to ensure accurate interpretation and age-appropriate recommendations. Future research should consider these results to ensure more accurate analyses and targeted policy recommendations. Furthermore, studies should explore the preferred PoD and the factors associated with achieving it.

## Data Availability

The original protocol can be found in PROSPERO (ID: CRD42024553349) https://www.crd.york.ac.uk/PROSPERO/view/CRD42024553349. All data generated or analyzed during this study are included in this published article.
