# Supplementary material for: Place of Death of People With Chronic Conditions in Latin America: A Systematic Review
Source: Public Health Rev. 2026 Mar 5;47:1609006. doi: 10.3389/phrs.2026.1609006 (PMC12999540; doi:10.3389/phrs.2026.1609006)
Supplement: Supplementary file 1 [file Table1.docx]

**Place of Death in Latin America: A Systematic Review**

**Supplementary material**

**S1.** Full query strings for searched databases.

| **Database** | **Query String** |
| --- | --- |
| EMBASE (OVIDSP) | "place of death" OR "site of death" OR "location of death" OR "place of dying").ab,ti  AND  ("Latin America" OR "South America" OR "Central America" OR  Argentina OR Bolivia OR Brazil OR Chile OR Colombia OR "Costa Rica" OR Cuba OR EcuadOR OR "Dominican Republic" OR "El SalvadOR" OR Guatemala OR Honduras OR Nicaragua OR Mexico OR Panama OR Paraguay OR Peru OR Uruguay OR Venezuela).ab,ti. |
| PsycINFO (OVIDSP) | ("place of death".ti,ab. OR "site of death".ti,ab. OR "location of death".ti,ab. OR "place of dying").af.  AND  ("Latin America" OR "South America" OR "Central America" OR  Argentina OR Bolivia OR Brazil OR Chile OR Colombia OR "Costa Rica" OR Cuba OR Ecuador OR "Dominican Republic" OR "El Salvador" OR Guatemala OR Honduras OR Nicaragua OR Mexico OR Panama OR Paraguay OR Peru OR Uruguay OR Venezuela).ab,ti. |
| Medline (OVIDSP) | ("place of death".ti,ab. OR "site of death".ti,ab. OR "location of death".ti,ab. OR "place of dying").af.  AND  ("Latin America" OR "South America" OR "Central America" OR  Argentina OR Bolivia OR Brazil OR Chile OR Colombia OR "Costa Rica" OR Cuba OR Ecuador OR "Dominican Republic" OR "El Salvador" OR Guatemala OR Honduras OR  Nicaragua OR Mexico OR Panama OR Paraguay OR Peru OR Uruguay OR Venezuela).ab,ti. |
| LILACS | tw:("place of death" OR "site of death" OR "location of death" OR "place of dying" OR  "lugar de muerte" OR "lugar de fallecimiento" OR "sitio de muerte" OR  "local de morte" OR "local do óbito" OR "local de óbito")  AND  tw:("América Latina" OR "América do Sul" OR "América Central" OR "Latin America" OR "South America" OR "Central America" OR  Argentina OR Bolivia OR Brasil OR Brazil OR Chile OR Colombia OR "Costa Rica" OR Cuba OR Ecuador OR "República Dominicana" OR "Dominican Republic" OR "El Salvador" OR Guatemala OR Honduras OR Nicaragua OR México OR Mexico OR Panamá OR Panama OR Paraguay OR Perú OR Peru OR Uruguay OR Venezuela |
| SciELO | "place of death" OR "site of death" OR "location of death" OR "place of dying" OR  "lugar de muerte" OR "lugar de fallecimiento" OR "sitio de muerte" OR  "local de morte" OR "local do óbito" OR "local de óbito"  AND  "Latin America" OR "South America" OR "Central America" OR "América Latina" OR "América do Sul" OR "América Central" OR  Argentina OR Bolivia OR Brasil OR Brazil OR Chile OR Colombia OR "Costa Rica" OR Cuba OR Ecuador OR "Dominican Republic" OR "República Dominicana" OR "El Salvador” OR Guatemala OR Honduras OR Nicaragua OR México OR Mexico OR Panamá OR Panama OR Paraguay OR Perú OR Peru OR Uruguay OR Venezuela |
